# Enterobacteriaceae producing extended-spectrum β-lactamases (ESBLs) colonization as a risk factor for developing ESBL infections in pediatric cardiac surgery patients: “retrospective cohort study”

**DOI:** 10.1186/s12879-017-2346-4

**Published:** 2017-03-29

**Authors:** Amine Cheikh, Bouchra Belefquih, Younes Chajai, Younes Cheikhaoui, Amine El Hassani, Amina Benouda

**Affiliations:** 1 Department of pharmacy, Abulcasis University, Cheikh Zaid Hospital, Rabat, Morocco; 2 Department of Microbiology, Faculty of Medicine, Mohammed VI University of Health Sciences, Microbiology Unit, National Reference Laboratory, Casablanca, Morocco; 3 Department of Intensive care, Abulcasis University, Cheikh Zaid Hospital, Rabat, Morocco; 4 Department of pediatric cardiac surgery, Cheikh Zaid Hospital, Rabat, Morocco; 5 Department of pediatrics, Mohammed V University, Cheikh Zaid Hospital, Rabat, Morocco; 6 Department of Microbiology, Cheikh Zaid Hospital, Rabat, Morocco

**Keywords:** Postoperative nosocomial infections, ESBLs, Cardiac surgery, Positive colonization

## Abstract

**Background:**

Children with cardiac defects need many hospitalizations and repetitive antibiotic therapies, with an increasing risk of colonization with multidrug-resistant bacteria (MDRB) such as extended-spectrum β-lactamase-producing Enterobacteriaceae (ESBL-E) Post-operative infections with these bacteria in paediatric cardiac surgery are life threatening. This article aims to study the prevalence of ESBL colonization among paediatric cardiac surgery patients, and to compare occurrence of post-operative infections with and without ESBL colonization. We also aim to study the correlation between the onset of postoperative infection and other parameters such as age, length of stay and preoperative antibiotic therapy.

**Methods:**

A retrospective cohort study included paediatric cardiac surgery patients in Cheikh Zaid hospital in Rabat, Morocco, between the 1st of January 2011 and 31 December 2014. A screening for ESBL colonization was requested for children who had a risk factor (previous hospitalization and/or taking antibiotics) at admission. Swabs were collected from three sites (throat, nose and anus). Two groups were compared – patients colonized and not colonized with ESBLs. Statistical analysis was performed using R software.

**Results:**

ESBL colonization screening was performed in 111 patients. Positive colonization was detected in 17 cases (15%). *Klebsiella pneumoniae (KP)*: 9 (53%) was the most frequently isolated species. Among the 17 patients, 23.5% (4/17) developed a postoperative infection due to ESBLs versus only one patient without colonization (1%).

There was a statically significant difference in terms of occurrence of postoperative infection between the two groups (*p* = 0.001). Relative risk of developing a postoperative infection with positive colonization was 22 (95% CI, 8.37–58.5).

**Conclusions:**

The analysis of colonization with multidrug-resistant bacteria and the prevention of nosocomial infections appear to be important challenges for paediatric cardiac surgery. Systematic screening of ESBL colonization for cardiac surgery could have a significant contribution, on one hand to guide prophylactic antibiotic therapy of patients, and on the other, to prevent spread of those infections.

## Background

The prevalence of ESBL-E carriage has changed significantly over time. However, this evolution varied by geographical region: it was more prevalent in the Western Pacific, Eastern Mediterranean, and Southeast Asia regions. In contrast, rates reported in Europe never exceeded 10% [1].

ESBL colonization among hospitalized patients worldwide is highly assessed. For instance, in 2003, the rate was about 11.8% in Spain [2], 16% in Lebanon [3] and 26% in Saudi Arabia [4]_._ Nevertheless, a prospective study performed in Turkey showed that 18.5% of children carried ESBL-producing *K. Pneumonia.* The impact rate of the nosocomial infections due to this strain is about 1.6% among hospitalized children [5].

One of the major causes of the increase in the prevalence of colonization by multidrug-resistant germs is the misuse of antibiotics. Antibiotic use creates a selective pressure on host bacteria in the large bowel, leading to the emergence of antimicrobial-resistant organisms, which in turn causes an increase in the number of carriers harbouring resistant bacteria and enhances the opportunity for these bacteria to cause infections [6].

In cardiac surgery, factors such as extracorporeal circulation, hypothermia, peripheral tissue hypo-perfusion and invasive medical devices like implantable materials may constitute major risks for increased perioperative infection and complication rates [7].

Whereas these infections are potentially lethal as well as costly, they remain in close relationship to catheter-related bloodstream infections in intensive care units (ICUs), which are a certain kind of medical practice [8, 9]. For instance, each year in the United States, central venous catheters are responsible for approximately 80,000 catheter-related bloodstream infections and, as a result, up to 28,000 deaths among ICU patients [10].

ESBL-producing organisms are responsible for a significant proportion of infections in ICUs. Treatment of these infections can place an added constraint on already overburdened health systems in developing countries [11]. Nosocomial infections attributable to these bacteria have been known to cause high mortality [12]. Several reports have addressed faecal colonization of these organisms during nosocomial infection outbreaks [13, 14].

Therefore, screening of ESBL colonization is very important in order to guide pre-operative prophylaxis and appropriate empirical antibiotic therapy of infected patients. In addition, this screening is useful to prevent nosocomial spread of these multi-drug resistant bacteria, reducing morbidity and mortality rates.

However, to our knowledge, there are few studies addressing the impact of carriage of ESBLs on the prevalence of postoperative infections in paediatric cardiac surgery either in Morocco or elsewhere.

This article aims to estimate the prevalence of ESBL colonization among children who underwent cardiac surgery in our hospital from January 2011 through December 2014. The primary objective of our study was to identify whether colonization with ESBLs is a risk factor for developing post-operative infections with these bacteria.

The secondary objective was to study the association between post-operative ESBL infection and parameters like age of patient, duration of hospitalization and pre-operative antibiotherapy.

## Methods

To identify whether colonization with ESBLs is a risk factor for developing post-operative infections with these bacteria, a retrospective cohort study was conducted at Cheikh Zaid hospital in Rabat, Morocco. The Cheikh Zaid hospital is an international teaching establishment. It contains 350 beds comprising various medical and surgical specialties, a paediatric unit, a maternity unit and three intensive care units with approximately 55,000 days of hospitalization and about 180 paediatric cardiac surgeries per year. Cheikh Zaid hospital is a reference centre for paediatric cardiac surgery at the national level.

Screening of ESBL colonization was performed based on risk criteria at admission (previous hospitalization and/or antibiotic therapy within 14 days before admission). Microbiological swabs were collected from the throat, nose and anus. They were spread onto two MacConkey Agar plates; one of the agars was supplemented with 1 μg/ml of cefotaxime and the other one with 1 μg/ml of ceftazidime, and both were incubated at 35 °C for a minimum of 24 h [15]. Suspected ESBL colonies were confirmed with the disc diffusion method on Mueller-Hinton Agar using cefotaxime, ceftazidime, cefpodoxime and ceftriaxone with amoxicillin/clavulanic acid as recommended by the European Committee on Antimicrobial Susceptibility Testing (EUCAST). Growing organisms were identified by using the API system (bioMérieux, Lyon, France) [16].

Data concerning post-operative infections were collected from laboratory information system and patient records. Only ESBL post-operative infections were reported in the present study.

Distribution of patients’ characteristics was reported using mean and standard deviation (SD) for quantitative variable, while qualitative data was reported using absolute frequencies and percentages. In the end, two groups were defined: ESBL colonized and non-colonized patients.

The prevalence of occurrence of ESBL post-operative infection was calculated for both groups. To compare qualitative variables, we used the Chi square test. When the application conditions were not met, we used Fisher’s exact test. The variables associated with postoperative infection (age, length of hospitalization and pre-operative antibiotherapy) were studied by univariate and multivariate logistic regression. The *p*-value was considered significant at a level lower than 0.05. Statistical analysis was performed using the R software environment and Statistical Package for the Social Sciences (SPSS) version 13.0 software for Microsoft Windows XP.

## Results

Screening for ESBL colonization was performed in 111 patients. The median age was 240 days (interquartile range [IQR]: 120–420 days). ESBL colonized patients represented 17/111 (15%) and non-colonized patients represented 94/111 (85%). Isolated species were *Klebsiella pneumoniae*: 9 (53%), *Escherichia coli*: 6 (35%) and *Enterobacter cloacae*: 2 (12%).

Among the 17 colonized patients, four (23.5%) developed a postoperative ESBL infection (*Klebsiella pneumoniae*: 2 (50%) and *Escherichia coli*: 2 (50%)). Among non-colonized patients, one patient (1%) developed an infection with ESBL (*Enterobacter cloacae*). The percentage of post-operative infection in colonized patients was 23.5% and it was 1% in patients who were non-colonized. The relative risk of developing a postoperative infection in colonized patients was 22 (95% CI, 8.37–58.5). There is a significant difference in terms of incidence of occurrence of ESBL postoperative infection between the two groups (*p* = 0.001). Univariate logistic regression showed that post-operative infection was not influenced by overall duration of hospitalization (*p* = 0.687), age (*p* = 0.753) or pre-operative antibiotherapy (*p* = 0.818). On the other hand, univariate linear regression showed that post-operative infection was influenced by carriage of ESBLs (*p* = 0.004). We did not perform multivariate analysis because only one parameter (ESBL colonization) was significant in the univariate analysis.

**Fig. 1:**
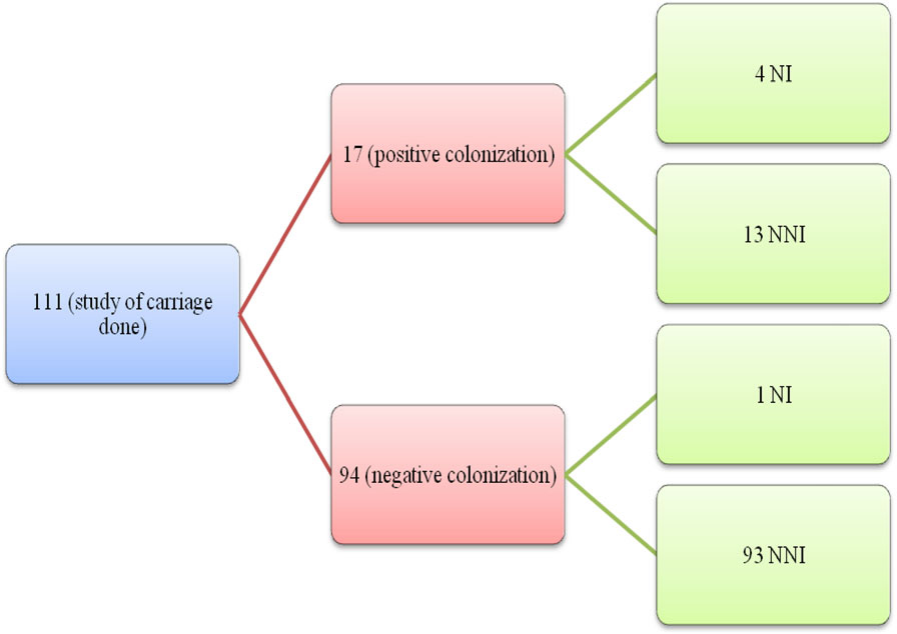
Description of the study design and results. (Legend: NI: Nosocomial Infection. NNI: No Nosocomial Infection)

The study design and results are detailed in Fig. 1 and Tables 1 and 2.

**Table 1:** ESBLs isolated in colonized patients who had post-operative ESBL infection

Patient	Colonization	Colonization bacteria	Site of Colonization	Infection bacteria	Site of infection	Type of surgery
1	Positive	*E. coli*	Nasal	*E. coli*	Blood	Aortic coarctation
2	Positive	*KP*	Nasal	*E. coli*	Pus/Wound	Isolated ventricular septal defect
3	Positive	*KP*	Anus	*K.P*	Pulmonary	Pulmonary atresia
4	Positive	*E. coli*	Anus	*E. coli*	Urinary	Tetralogy of Fallot

*E. coli Escherichia coli, KP Klebsiella pneumonia, EC Enterobacter cloacae*

**Table 2:** ESBLs isolated in colonized patients who did not have post-operative ESBLs infection

Patient	Colonization	Colonization bacteria	Site of Colonization	Type of surgery
1	Positive	*KP*	Anus	Pulmonary atresia
2	Positive	*E. coli*	Nasal	Atrioventricular septal defects
3	Positive	*E. coli*	Anus	Atrioventricular septal defects
4	Positive	*KP*	Anus/Throat	Atrioventricular septal defects
5	Positive	*E. coli*	Urinary tract	Pulmonary artery banding
6	Positive	*EC*	Anus	Pulmonary artery banding
7	Positive	*EC*	Anus	Isolated ventricular septal defect
8	Positive	*KP*	Nasal/Anus	Isolated ventricular septal defect
9	Positive	*KP*	Nasal	Isolated ventricular septal defect
10	Positive	*KP*	Anus	Aortic coarctation
11	Positive	*KP*	Anus	Aortic coarctation
12	Positive	*KP*	Throat	Aortic coarctation
13	Positive	*E. coli*	Nasal/Anus	Complete transposition

*E-coli Escherichia coli, KP Klebsiella pneumonia, EC Enterobacter cloacae*

## Discussion

According to our knowledge, few studies have focused on post-operative infections caused by ESBLs in paediatric cardiac surgery, especially with regards to previous colonization with ESBLs. No data is available for ESBL colonization and/or infection in children with congenital heart disease in Morocco.

In our country, some studies showed that the occurrence of ESBLs was high on faecal carriage (42.8%) [17], 28.6% in blood culture, superficial and deep pus [18] and catheters and 20.7% in blood cultures, urine, pus and bronchial sampling [19].

Multidrug-resistant bacteria (MDRB) screening is recommended as a standard protocol for all cardiac surgery patients [20]. Kim et al. [21] suggest that in a non-epidemic situation, systematic detection of ESBLs in ICU patients is not cost-effective, and that stringent contact precaution for infected patients might be adequate. However, our institution applies selection criteria based on previous hospitalization or prior use of antibiotics to perform MDRB screening. Screening of all MDRBs, including MRSA and carbapenemases, was performed for all patients with a risk criterion. In this study, we only reported ESBL-E screening because no MRSA strain was isolated and only one strain of *Klebsiella pneumoniae* was both carbapenemases- and ESBL-producing. Therefore, it appears more relevant to focus on ESBLs.

We found 15% ESBL colonization, similar to a Polish study on the same population (16% MDRB) [20] and to a reported colonization rate in a 2012 report from France (15%) [22]. However, it was lower than those reported by Kim et al. in a Korean study of intensive care unit patients (28.2%) [21]; in another recent Korean study, the authors reported a rate of 42.5% in ICU patients and 20.3% even in healthy persons [23].

The majority of ESBL strains in this study were isolated from anal swab. This finding is similar to those reported in colonization screening by other authors [20]. It could be interesting to discuss whether screening for MDRB in our setting should be done only with anal swab (just one site of sampling), with the objective of enhancing the cost-effectiveness of MDRB screening. The most common isolated species were *Klebsiella pneumoniae* in our study, while others reported higher frequencies of *Escherichia coli* isolation [20].

Otherwise, nosocomial infections in children after heart surgery occur in 12.9% to 30.8% of cases [24, 25]. Bloodstream infections (BSI) are one of the most common infections [26–28] seen in children undergoing cardiac surgery, with studies reporting incidence as high as 65% of total nosocomial infections [25].

The most common organisms detected in infectious endocarditis after surgery for congenital heart defect were *Streptococcus viridans* and *Staphylococcus aureus* (each 23% of total), as reported by Cynthia et al. [29]. However, other studies have reported that Gram-negative bacilli (GNB) were the most frequent isolates in nosocomial pneumonia (NP) in infants in a paediatric surgical ICU after cardiac surgery (86.1%) [30]. The main GNB were *Acinetobacter baumannii* isolates (13.9%), *Pseudomonas aeruginosa* (10 isolates, 12.7%), *Klebsiella pneumoniae* (7 isolates, 8.9%), and *E. coli* (6 isolates, 7.6%). The bacteria-producing extended spectrum beta-lactamases were mainly *K. pneumoniae* and *Escherichia coli* [30].

In our study, infection with ESBLs was found in five patients, four of them likely infected with a strain of the same species isolated upon colonization screening at admission, with the same antibiotic susceptibility features. We could not confirm the clonality of colonization and infection isolates with molecular study due to lack of resources. Only one case was infected with *E. coli* after being colonized by *K. pneumoniae*. This case was probably a nosocomial infection contracted during hospitalization in the ICU. We could not confirm this due to a lack of techniques and reagents. In addition, the conventional technique used for the detection of ESBL-E is not very sensitive; possible colonization by *E. coli* could not be identified with this technique. On the other hand, since this case was a newborn, a possible carriage in the mother could be at the origin of this contamination with *E. coli*.

Carriers of ESBLs appear to be at higher risk of subsequent bacteraemia compared with non-carriers [31]. Due to the spread of extended-spectrum β-lactamase-producing *E. coli* (ESBL-EC) carriage, a practitioner may be tempted to modify empirical antibiotic therapy in known carriers with signs of infection.

Our results, even with a lower rate (23%), demonstrate that previous colonization with ESBLs may be a risk factor for the occurrence of infections after cardiac surgery in paediatric patients. In fact, the difference in the incidence of those infections between the two groups (colonized and non-colonized) was significant.

So, should we prescribe antibiotic prophylaxis systematically on previously colonized children undergoing cardiac surgery?

Actually, antibiotic prophylaxis for cardiac surgery is a controversial area. Recent systematic reviews and meta-analyses of randomized controlled trials have concluded that surgical site infection can be reduced by prolonging prophylaxis for 24–48 h. In addition, post-operative pneumonia and every cause of mortality can be reduced by giving agents with both anti-Gram-negative and anti-Gram-positive activity [32]. The choice of the most appropriate regimen remains open to debate, and yet no recommendations exist for paediatric cardiac surgery patients.

Our goal was to gain an insight into occurrence rate of colonization and infection with ESBLs in this specific population. Our findings were very concerning, and may influence our screening and antibiotic prophylaxis policies.

It is clear that infection control for ESBL-producing strains would be achieved through active surveillance of high-risk patients, such as those in the ICU [33]. Some authors claim that strict contact precautions might be more appropriate and cost-effective than systematic MDRB screening. Nevertheless, ESBL colonization rate in paediatric cardiac surgery patients is still relatively low, allowing the prevention of infection through systematic screening. This screening will provide epidemiological data to guide antibiotic therapy policies. It must be applied for all MDRBs, even if ESBLs are the most common.

In fact, besides ESBLs, carbapenemase-producing *Enterobacteriaceae* seem to be of great concern in our geographic area. Their recent emergence and the potential for horizontal transfer of resistance genes make critical the control of their spread while the frequencies are still low [21]. This will require rapid and accurate testing of carbapenem-resistant isolates from clinical specimens, as well as the adoption of appropriate infection control procedures if carbapenemase-producing isolates are found. In addition, the incidence of CRE should be monitored continuously. If CRE incidence increases, more aggressive management, such as active surveillance of high-risk patients, will be necessary [21].

Our study has many limits. Due to the retrospective design, we could not have all clinical data that would be of interest with regard to colonization and infection with ESBLs, such as hospitalization duration before surgery, mortality, detailed antibiotic regimen before and after surgery, and other infections apart from ESBL infections.

## Conclusions

Our study demonstrated that the positive carriage of ESBLs is a risk factor for post-operative infection in paediatric cardiac surgery patients. The screening for these bacteria allows the isolation of patients and the prevention of possible cross-contamination between highly at-risk patients such as our study population. Significant progress in the field of hospitalization conditions, hygiene and hand washing will help us to reduce significantly the prevalence of ESBL transmission and post-operative infection in our context.

## Abbreviations

BSI: Bloodstream infections
CPE: Carbapenemase-producing Enterobacteriaceae
CRE: Carbapenem-Resistant Enterobacteriaceae
ESBL-E: Extended-spectrum betalactamase producing–Enterobacteriaceae
GNB: Gram-negative bacilli
ICU: Intensive Care Unit
MDRB: Multidrug resistant bacteria
NI: Nosocomial infections
NNI: Non nosocomial infections
NP: Nosocomial pneumonia
